# Extracellular Vesicles – Biomarkers and Effectors of the Cellular Interactome in Cancer

**DOI:** 10.3389/fphar.2013.00021

**Published:** 2013-03-06

**Authors:** Janusz Rak

**Affiliations:** ^1^The Research Institute of the McGill University Health Centre, Montreal Children’s Hospital, McGill UniversityMontreal, QC, Canada

**Keywords:** extracellular vesicles, exosomes, oncogenes, cancer, cellular interactions

## Abstract

In multicellular organisms both health and disease are defined by patterns of communication between the constituent cells. In addition to networks of soluble mediators, cells are also programed to exchange complex messages pre-assembled as multimolecular cargo of membraneous structures known extracellular vesicles (EV). Several biogenetic pathways produce EVs with different properties, and known as exosomes, ectosomes, and apoptotic bodies. In cancer, EVs carry molecular signatures and effectors of the disease, such as mutant oncoproteins, oncogenic transcripts, microRNA, and DNA sequences. Intercellular trafficking of such EVs (oncosomes) may contribute to horizontal cellular transformation, phenotypic reprograming, and functional re-education of recipient cells, both locally and systemically. The EV-mediated, reciprocal molecular exchange also includes tumor suppressors, phosphoproteins, proteases, growth factors, and bioactive lipids, all of which participate in the functional integration of multiple cells and their collective involvement in tumor angiogenesis, inflammation, immunity, coagulopathy, mobilization of bone marrow-derived effectors, metastasis, drug resistance, or cellular stemness. In cases where the EV role is rate limiting their production and uptake may represent and unexplored anticancer therapy target. Moreover, oncosomes circulating in biofluids of cancer patients offer an unprecedented, remote, and non-invasive access to crucial molecular information about cancer cells, including their driver mutations, classifiers, molecular subtypes, therapeutic targets, and biomarkers of drug resistance. New nanotechnologies are being developed to exploit this unique biomarker platform. Indeed, embracing the notion that human cancers are defined not only by processes occurring within cancer cells, but also between them, and amidst the altered tumor and systemic microenvironment may open new diagnostic and therapeutic opportunities.

## Introduction

It could be argued that the ultimate test for the correctness and utility of concepts explaining the nature of human neoplasia is not just their intellectual sophistication, but rather the robustness of therapeutic predictions they inspire. In this regard the justified enthusiasm over the early experiences with targeted anticancer agents is increasingly mixed with a sense of surprise that biological agents designed to strike at, what is often seen as the very heart of the malignant process (oncogenic pathways and driver mutations), frequently deliver only partial or temporary effects, rather than being curative (Murdoch and Sager, 2008).

These limitations could in each case be explained by several detailed biological mechanisms, many of which are intensively studied (Broxterman et al., 2003; Bergers and Hanahan, 2008). However, a part of this conundrum could also lie in the possibility that some major pieces of cancer pathogenesis puzzle are still missing, or have been overshadowed by technological developments, often on the expense of concepts. In this regard there seem to be at least two self-imposed barriers in the current thinking about the more effective ways to execute anticancer therapy. First, there is a tendency to grapple with the uncomfortable complexity of human cancers through attempts to seek simplicity in their common denominators, such as those exemplified by the “hallmarks of cancer” (Hanahan and Weinberg, 2011). While such organizing principles can be invaluable in the conceptual, fundamental and educational sense, they also downplay profound distinctions between disease states that have traditionally been classified as “cancer,” and are effectively vastly different from one another. It is unlikely that effective treatment of, say glioblastoma (GBM) could be derived from first principles that operate above this diversity.

Second, the fascination with the technological ability to detect and catalog tangible changes in the genome and epigenome of cancer cells, sometimes leads to the notion that these intracellular events play a causative role in the disease, because of their selective enrichment (dominance) in the cancer cell population (Vogelstein and Kinzler, 2004; Avraham and Yarden, 2011; Flaherty et al., 2012). This approach has led to spectacular developments in the area of targeted anticancer therapeutics. It also brought the sense that cancer can essentially be explained (in both general terms and in detail) by inherent nature of various molecular intracellular alterations, by virtue of their subsequent Darwinian selection through competition of affected cancer cells, or stem cells (Nowell, 1976; Greaves and Maley, 2012). Consequently, the alternative approaches of peering through the nebulous questions surrounding “cancer cell societies,” their complexity, heterogeneity, and inner dynamics (Heppner, 1989; Miller and Heppner, 1990) largely fell out of favor, wherein may lay some of the currently encountered limitations.

## Complexity of Human Cancers

The advent of molecularly based, targeted drugs brought the hard scientific rationality into the area of oncology practice (Druker, 2004). Perhaps, the most impressive examples of this invaluable and lasting impact include the introduction of imatinib into therapy of chronic myelogenous leukemia (CML), trastuzumab into breast cancer (BCa), sunitinib into renal cancer (RCC), avastin into colorectal tumors (CRC), and vemurafenib into melanoma (CMM; Druker, 2004; Flaherty et al., 2012), all of which continue to stimulate hope and well justified excitement.

On the other hand there is a growing realization that even within the restrictive subgroups of cancers the disease cannot necessarily be reduced to intracellular iterations of mutations and the related epistatic signaling defects amenable to suppression by the respective pharmaceuticals. Indeed, the major genomic and gene expression profiling projects (e.g., TCGA) have documented the existence of unsuspected multiplicity of molecular disease variations, defined by mixtures of prevalent and rare mutations, the genesis, and causative role of which is not always immediately obvious (Phillips et al., 2006; Wood et al., 2007; Curtis et al., 2012). Moreover, the recent progress in the next generation sequencing technologies (NGS) revealed an even more limitless, evolving, and highly regional genetic heterogeneity, including cellular diversity between and within individual lesions in the same cancer patient (Gerlinger et al., 2012). Indeed, at least in certain disease settings, distinct genetic defects with a putative disease-propagating potential seem to exist within each tumor cell, or in their subsets, the myriads of which may populate tumor masses and distant sites in individual cancer patients (Turner and Reis-Filho, 2012). Such intricacies have long evaded traditional molecular profiling efforts, and they clearly make molecular targeting, and individualized cancer care more difficult to conceptualize (as each patient may harbor multiple disease causing mechanisms).

On the other hand, findings revealing the cellular complexity of various cancers tend to revive an interest in the actual meaning, causes, and consequences of tumor heterogeneity, and they pave the way to studies on the evolution of, and phylogenetic linkages within, the hierarchical cancer cell subpopulations (Dick, 2008; Greaves and Maley, 2012; Wu et al., 2012). There is an emerging realization that *functional units* of various cancer-related processes (e.g., stemness, metastasis, angiogenesis) could be pre-programed within multicellular assemblies rather than in phenotypes of individual cancer cells (Rak, 2006; Greaves and Maley, 2012). This alternative optics sees cancer cell populations as complex systems, and challenges the traditional tenets of cancer genetics, by placing considerable emphasis on low frequency events, cryptic cellular subsets, and cooperation rather than domination of specific cellular clones, all of which may contribute to certain aspects of the disease (Wood et al., 2007; Gerlinger et al., 2012; Wu et al., 2012).

For example, the concept of “driver mutations” is largely based on statistical frequency of certain genetic events in the cancer cell population, and their presumed selective (competitive) and cell-intrinsic advantage. This is often taken as tantamount to playing a causative and pathogenetic role in a particular cancer. However, such mutational preponderance may also reflect the history rather the driving mechanism of the disease. Indeed, the propensity of a particular gene to mutate early on in the disease process (hence commonality) may not necessarily prove that the mutation in question is indispensable at the later stages of cancer progression. At the very least, the roles of mutational events may be more complex than can be inferred from their statistical frequencies and may encode not only cell-autonomous traits, but also define patterns of intercellular interactions.

A case in point could be the frequent oncogenic amplifications of the epidermal growth factor receptor (EGFR), or the related oncogenic mutation (EGFRvIII) in GBM (Wen and Kesari, 2008). It is thought provoking that mutant EGFR is not sufficient to cause glioma in animal models (Huse and Holland, 2009). Moreover, pharmacological targeting of EGFR provides a relatively modest therapeutic benefit, and only to GBM patients with certain configuration of other molecular changes, such as intact expression of the PTEN tumor suppressor gene (Mellinghoff et al., 2005). Notably, GBMs often contain stably mosaic cellular compositions, including the concomitant presence of tumor cell subsets with and without EGFRvIII mutation, or co-existing tumor micro-regions harboring cell subpopulations with oncogenic amplifications of either *EGFR* or *PDGFR* genes (Biernat et al., 2004; Ramnarain et al., 2006; Inda et al., 2010; Snuderl et al., 2011). This sustained (active) heterogeneity is not readily detectable by profiling studies and the reasons why it exists are not always immediately obvious. Nonetheless, the stable co-existence of genetically distinct cell subsets suggests that their compositions (cooperation) may be a source of aggressive disease, and confers a collective, rather than individual, growth advantage.

It is also of note that the seemingly permanent, selective, and presumably advantageous genetic alterations (e.g., EGFR amplifications), are often lost from glioma cells as soon as these cells are separated from each other and placed in cell culture (Bigner et al., 1990; Schulte et al., 2012). Regardless of the underlying causes and mechanisms of this intriguing change, the loss of genetic mutation upon transfer into a different microenvironment (e.g., *in vitro*) suggests that it is the communication with pericellular surroundings (*in vivo*) of these malignant cells that may ultimately control the status and prevalence of their cell-intrinsic “driver mutations.”

## Intercellular Communication in Cancer Progression

Revesz (1956) described an experiment involving injection into syngeneic mice of either small numbers of viable cancer cells (10^4^), or much larger numbers (10^8^) of the same cells that have been previously rendered mitotically dead through the exposure to a high dose of ionizing radiation. While neither of these inoculations produced tumors for up to 90 days, they did so when combined into one injection (Revesz, 1956). In today’s terms this experiment (*Revesz effect*) may suggest that even under the best of conditions the viable tumor initiating cells (TICs) require a supportive niche, which could consist of the remaining, even mitotically dead, or otherwise non-tumorigenic cancer cells, as well as other stromal elements. Although the irradiated cells were, by definition, devoid of TIC properties, they clearly played other biological roles by *interacting* with viable TICs in this setting, a process that could be linked to paracrine interactions, immune-protection, or angiogenesis (Rak, 2006).

There are numerous examples of the influence cell-cell interactions may exert over the malignant potential of individual cancer cells, or their subsets. For example, in their now classical experiments, Mintz and Illmensee isolated normal mouse embryonic stem (ES) cells and demonstrated that they form aggressive teratomas upon ectopic (subcutaneous) inoculation. Remarkably, these teratoma cells were able to give rise to normal mice upon their incorporation into blastocysts, even after repeated passage as tumors in mice, for several years (Mintz and Illmensee, 1975). Similarly, Bissell and colleagues discovered that Rouse sarcoma virus (RSV) was unable to transform avian embryos, but could cause cancer in analogous chick tissues when injected post birth (Dolberg and Bissell, 1984). More recently, normal neuronal stem cells were found to come into contact with, and influence, their malignant GBM counterparts, at least in part through stimulation of the vanilloid receptor (TRPV1; Stock et al., 2012).

However, there is probably no better example of the role cellular interactions play in cancer then the process of metastasis. The “seed and soil” relationship between metastatic cancer cells and their organ destinations involves multiple and reciprocal signals, which are of either selective or instructive nature. Different populations of cancer cells colonize specific organ sites in a process now known to be influenced not only by the cancer cell genome, but also by the host genetic background and tissue microenvironment (Paget, 1889; Fidler, 2003; Hunter, 2006; Chiang and Massague, 2008). In invasive tumors stromal cells may acquire genetic mutations (Hida et al., 2004; Hill et al., 2005). Both the vasculature and bone marrow-derived cells (BMDCs) that infiltrate distant organ sites are involved in the execution of the metastatic niche program (Folkman and Kalluri, 2003; Kaplan et al., 2005; Peinado et al., 2012). In some instances only a small (normally undetectable) proportion of cancer cells may contain genetic determinants associated with the metastatic process, and these signatures are only revealed (enriched) upon tumor dissemination to distant sites (Gerlinger et al., 2012; Wu et al., 2012). It is possible that in these settings the rare mutant genes confer interactive phenotypes unique to the sites of metastasis.

The genetic evolution of cancer cell clones during disease progression and metastasis is often viewed mainly as a result of competitive interactions, resulting in the selection of specific and advantageous cellular traits (Nowell, 1976; Vogelstein and Kinzler, 2004). However, as early as in 1980s Heppner, Miller and their colleagues begun exploring the possibility that, at least under certain circumstances, some of the key events in cancer can be defined by cellular composition and intercellular cooperation, rather than being solely an outcome of a Darwinian dominance (Heppner, 1989). Using a unique series of sister cancer cell lines derived from a single murine mammary tumor, these investigators demonstrated the cooperative (interactive) paradigm applies to such diverse biological processes as tumor growth, metastasis, and drug resistance (Miller and Heppner, 1990). Similar findings have been documented in more recent studies, again emphasizing that molecular cell-cell interactions may occur over short and long distances, and may include paracrine and inflammatory components (McAllister et al., 2008).

A particularly elegant example of cellular interrelationships in cancer is described in a recent study by Wu and colleagues. These investigators embarked on the analysis of the role of the oncogenic *Ras* mutation (*RasV12*) and the loss of *scribbled* tumor suppressor (*scrib-*) in the context of ocular tumor development in *Drosophila*. Interestingly, each of these genetic defects alone was found to be insufficient to trigger the overt eye disease, but their co-existence in the same ocular cell resulted in formation of aggressive tumors, thereby revealing the expected genetic cooperation. Perhaps less expected, but fascinating, was the fact that tumors did emerge even when *RasV12* and *scrib*− mutations occurred in separate cellular subsets within the same eye. This suggests that genetically altered cellular subpopulations may cooperate in forming a pro-tumorigenic microenvironment, a “critical mass” required for the onset of the overt disease (Wu et al., 2010). These and other examples raise the possibility that intercellular exchange of information (influenced by genetic defects) may play a significant, perhaps a defining, role in the context of at least some cancers. If this is the case, the content of molecular information required for cellular cooperation and the nature of processes leading to such intercellular exchanges, integration, and dialog could be of considerable diagnostic and therapeutic significance.

## Extracellular Vesicles as Mediators of Multicellular Integration

Interactions between cancer cells and their surroundings are multifaceted. While the related analyses usually concentrate on cellular secretome, and molecular recognition events involving soluble or cell-associated ligands and their receptors, there are several other levels of intercellular communication worthy of consideration. Thus, adjacent cells may enter into a deeper level of functional integration through physical contact and/or exchange of cellular fragments, a process which may lead to “sharing” of more complex integral segments of molecular machinery of a given cell. Such exchanges have been described upon formation of intercellular junctions, synapses, cytonemes, or by membrane swapping processes known as trogocytosis, all of which mostly operate over short distances (Roy et al., 2011).

In this regard, a unique form of molecular exchange that can operate over both, short or long distances consists of shedding and uptake of extracellular vesicles (EVs). EVs are plasma membrane structures that emanate from all cells, especially upon cellular stress, activation, or transformation (Thery et al., 2009; Record et al., 2011; Kalra et al., 2012). Such vesicles originate either at cell surfaces (ectosomes), or within the endosomal pathway (exosomes), the elements of which may subsequently relocate to the inner portion of the plasma membrane and release exosomes into the extracellular space. Moreover, vesiculation may also accompany apoptotic cell death, in which case it is a terminal, but biologically important, process that may influence surrounding viable cells in several ways, including transfer of mutant, oncogenic DNA, and stimulation (Bergsmedh et al., 2001).

It should be emphasized that the abundance, heterogeneity, and molecular characteristics of EVs reflect not only the identity and state of their parental cells, but also the diversity of the underlying biogenetic pathways (Thery et al., 2009). Thus, exosomes are usually relatively small (30–100 nm) and upon centrifugation sediment within a defined sucrose gradient density (1.11–1.20 g/ml). They are rich in tetraspanins (e.g., CD63, CD81, CD9), and their production is controlled by several emerging regulatory mechanisms, including elements of the endosomal sorting complex required for transport (ESCRT), Rab proteins (e.g., Rab27a), p53/TSAP6 pathway, ceramide, and neutral sphingomyelinase, to mention the most studied effectors (Yu et al., 2006; Lespagnol et al., 2008; Trajkovic et al., 2008; Ostrowski et al., 2010; Bobrie et al., 2012; Peinado et al., 2012).

Membrane-derived ectosomes (microparticles) are normally much larger (100–1000 nm) then exosomes, and they may contain cell lineage markers, cell surface receptors, and often (but not always) abundance of phosphatidylserine (PS) residues. Their production is regulated by a distinct set of molecular mechanisms including AKT activation, acidic sphingomyelinase, intracellular calcium fluxes, and enzymes involved in the maintenance of membrane phospholipid asymmetry (Piccin et al., 2007; Bianco et al., 2009; Di Vizio et al., 2009; Thery et al., 2009). In cancer, ectosome-like structures may originate from membrane blebs that are associated with the ameboid motility of certain types of tumor cells. These abnormal EVs are often referred to as large oncosomes (Di Vizio et al., 2009).

The emerging evidence points to several additional pathways involved in cellular vesiculation, the strict assignment of which to exosome, ectosome, or oncosome production is not always clear. This includes EGFR, Ras, RhoC/ROCK, DRF3, Arf6, Rap2b, MEKK2, and other signaling modules (Greco et al., 2006; Di Vizio et al., 2009; Muralidharan-Chari et al., 2009; Cronan et al., 2012; Li et al., 2012). Given the link between many of these regulators and processes that control cellular growth, survival, and motility, it is not surprising that oncogenic pathways, such as those triggered by mutant EGFR, MET, K-ras, AKT, and the loss of tumor suppressors, including p53 or PTEN, influence the nature and composition of tumor-derived EVs. It is possible that the emission of EVs represents one of several ways in which oncogenic transformation re-programs the patterns of intercellular interactions, to serve as a mechanism that conditions tissue microenvironment to promote (or curtail) neoplastic growth (Rak and Guha, 2012).

Production of EVs is thought to fulfill different basic cellular functions. In some instances cells simply dispose of the spent or harmful molecular content in this manner, as is the case for transferrin receptors during erythrocyte maturation, as well as during vesicular shedding of complement complexes, or pro-apoptotic caspase 3 (Johnstone, 2006; Abid Hussein et al., 2007). EVs may also play a role in modulation of cellular signaling patterns, e.g., by “deporting” certain activated modules to attenuate their intracellular regulatory influence. This has been postulated to occur in the case of EGFR, beta-catenin, or viral LMP1 proteins (Chairoungdua et al., 2010; Verweij et al., 2011; Garnier et al., 2012). Moreover, the EV-mediated release of certain membrane-associated ligands, such as the Notch agonist, delta like 4 (Dll4), may change the signaling characteristics of the respective pathways (e.g., from stimulation to inhibition) and in an autocrine, or paracrine manner (Sheldon et al., 2010). Exosomes may also enable formation of morphogenetic gradients of various mediators required for organ development, as suggested by the recent studies on Wnt (Gross et al., 2012). They may also contribute to tumor-stromal interactions (Ghosh et al., 2010; Luga et al., 2012). In a broader sense, circulating EV may act as important systemic carriers of molecular information and be involved in modulation of the immune, inflammatory, hemopoietic, and hemostatic functions. Consequently, null mutations within the exosomal pathway often lead to anemia, bleeding, and immunological complications (Piccin et al., 2007; Tolmachova et al., 2007; Lespagnol et al., 2008; Thery et al., 2009).

The ability of EVs to selectively assemble multiple bioactive molecules, such as signaling and regulatory proteins, lipids, mRNA, microRNA, and DNA sequences, carries an intrinsic potential to transfer of this material to various recipient cells capable of the efficient EV uptake (Ratajczak et al., 2006; Valadi et al., 2007; Thery et al., 2009). For these reasons EVs are sometimes referred to as intercellular “signalosomes” (Record et al., 2011). This property is often seen as a unique pathway of molecular and functional integration and modulation of multicellular processes. EVs act across cellular boundaries and make the respective molecular regulators available to cells that normally do not express them endogenously, or do so at low levels. We propose that this circumstance may assume a particular significance in the context of cancers.

## The Emerging Role of Extracellular Vesicles in Cancer

**Figure 1:**
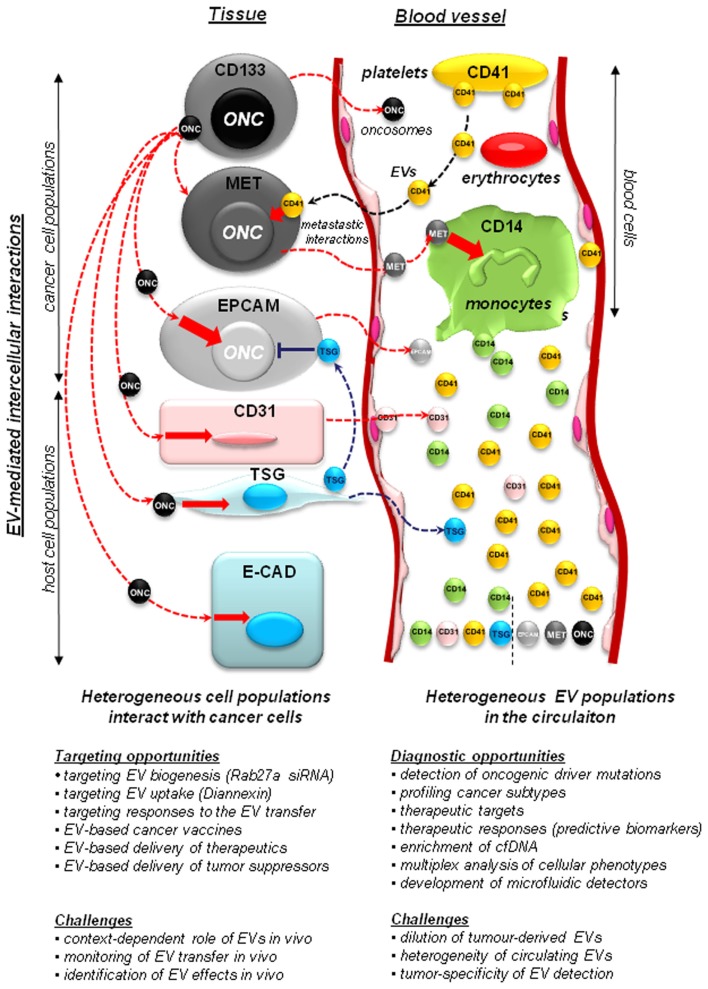
**Implications of the vesiculation process in cancer**. Heterogeneous populations of cancer and host cells remain functionally interconnected. This process is, at least in part, mediated by extracellular vesicles (EVs) that shuttle molecules between different populations of cancer cells and between them and the non-transformed host stromal and blood cells. Cancer cells may include stem cell-like compartment (e.g., cells expressing CD133), or cells that express other surface molecules (MET, EGFR, EPCAM – see text) with important functions. Some of the more notable EV-mediated molecular exchanges described recently in the literature include: (i) transfer of oncogenes (ONC) from cancer cells to normal cells, a possible horizontal transformation of these cells resulting in changes in phenotype, as well as (in some cases) acquisition of tumorigenic properties; (ii) transfer of tumor suppressors (TSG), such as PTEN between cells, with a possible impact on negative control of cellular transformation; (iii) transfer of MET receptors from metastatic cancer cells to myeloid cells (CD11b+) to modulate metastatic niche effects; (iv) contribution of platelet-derived EVs (CD41+) to the metastatic phenotype of cancer cells, and several other effects; (v) shedding of EVs by activated endothelium (CD31+). While tumor-derived EVs constitute a minority within the pool of particles circulating in plasma, they can be detected for diagnostic purposes. The diagnostic and therapeutic opportunities, and challenges associated with studies on cellular vesiculation are described in the text in detail.

Cancer cells exhibit an altered, and usually pronounced, tendency to produce EVs (vesiculate). This may be reflected by numbers of emitted EVs, their sizes, structures, overall protein content, and the composition of their molecular cargo (Al-Nedawi et al., 2008; Di Vizio et al., 2009; Palma et al., 2012; Peinado et al., 2012). Both tumor cells and their activated host counterparts (macrophages, fibroblasts, platelets) may release EVs into the tumor microenvironment and circulating biofluids (e.g., blood, lymph, or ascites; Figure 1). This creates a hitherto unappreciated potential for the intercellular exchange of bioactive cancer-related macromolecules (both soluble and cell-associated), between ostensibly distinct cell types. This process that has recently emerged as an important modulator of the antitumor immunity, inflammation, angiogenesis, thrombosis, invasion, metastasis, resistance to therapy, and other key biological events (Andre et al., 2004; Dolo et al., 2005; Taylor and Gercel-Taylor, 2005; Gesierich et al., 2006; Ratajczak et al., 2006; Bebawy et al., 2009; Camussi et al., 2010; Hendrix et al., 2010; Hood et al., 2011; Zwicker et al., 2011; Peinado et al., 2012).

A unique aspect of the EV production by cancer cells is related to the extracellular emission and intercellular trafficking of molecules containing oncogenic mutations (Al-Nedawi et al., 2008; Rak and Guha, 2012). This includes activated oncoproteins, their transcripts, oncogenic DNA sequences, and oncogenic, as well as regulatory micro RNA (oncomiRs; Al-Nedawi et al., 2008; Skog et al., 2008; Holmgren, 2010; Pegtel et al., 2010; Balaj et al., 2011; Demory et al., 2012; Garnier et al., 2012). Indeed, studies performed in a number of experimental systems suggest that oncogene-containing EVs (oncosomes) can mediate intercellular trafficking of mutant molecules, once thought to be strictly intracellular. The uptake of this transforming cargo by indolent or normal recipient cells was found to cause changes in their phenotype and biological behavior. For example, the uptake of oncogenic EGFR (or EGFRvIII) mediated by this mechanism causes an apparent phenotypic transformation of indolent glioma cells, and leads to reprograming of growth factor pathways in normal endothelial cells (Al-Nedawi et al., 2008, 2009; Skog et al., 2008).

Other oncogenes, such as Ras, Myc, SV40 LT, LMP1 were also reported to traffic between cells as cargo of various types of EVs, whereupon they induce signaling and gene expression effects across cellular boundaries and often at a distance from their cellular sources (Bergsmedh et al., 2001; Balaj et al., 2011; Demory et al., 2012; Verweij et al., 2012; Meckes et al., 2010). Conversely, the discovery of EV-mediated transfer of tumor suppressors, such as PTEN, has led to a suggestion that such mechanism may curtail the aggressiveness of cancer cells, which may be able to take up this material from their indolent, or non-transformed and PTEN-proficient counterparts (Lee et al., 2011; Putz et al., 2012).

It should be mentioned that the effects caused by the uptake of oncogenic proteins (or nucleic acids) may vary considerably in different cellular contexts. In some instances the recipient cells undergo a relatively transient phenotypic transformation, or exhibit accelerated growth rate, clonogenicity, and proangiogenic activity (Al-Nedawi et al., 2008). However, the uptake of apoptotic bodies containing mutant genomic sequences encoding *H-ras*, *Myc*, or *SV40 LT* (DNA) have been reported to cause an overt tumorigenic conversion of immortalized fibroblasts (Bergsmedh et al., 2001). Similarly, permanent changes sufficient to drive tumor growth *in vivo* were also described in fibroblasts subjected to the EV-mediated transfer of tumor cell-derived tissue transglutaminase and fibronectin (Antonyak et al., 2011).

Moreover, the intercellular vesicular trafficking of various bioactive molecules has long been implicated in metastasis (Poste and Nicolson, 1980; Janowska-Wieczorek et al., 2005; Hao et al., 2006; Jung et al., 2009; Hood et al., 2011; Peinado et al., 2012). A particularly striking recent example in this regard entails the ability of metastatic melanoma cells to emit exosomes containing high levels of the MET proto-oncogene. The uptake of this receptor by host BMDCs leads to their stable “re-education,” whereby they become conditioned to support formation of pre-metastatic niches in target organs to which melanoma cells can subsequently lodge (Peinado et al., 2012). Interestingly, an avid uptake of MET by myeloid cells is not only observed in experimental settings, but also occurred in stage IV melanoma, as indicated by the high levels of MET staining on the surface of the circulating BMDCs in these patients (Peinado et al., 2012).

Tumor-derived EVs may also be enriched in clusters of non-mutated, but otherwise biologically active regulatory proteins. Their composition may be reflective of the identity and state of their parental cells, and could possess diagnostic, as well as functional significance, for various aspects of disease progression. This includes wild type growth factor receptors (EGFR, HER-2, or MET), procoagulant receptors (tissue factor), heat shock proteins (HSP70), angiogenic molecules (IL-8), proteolytic enzymes (MMPs), and other bioactive cargo (Rak and Guha, 2012).

While the role of vesicles in the intercellular trafficking of oncogenic and tumor promoting molecules is highly intriguing, the indispensability of this process during progression of human cancers remains unclear, and is likely context-specific. For example, in the model of A431 carcinoma a PS-blocking agent, Diannexin, inhibits the uptake of tumor cell-derived EVs by endothelium. While this leads to antitumor and antiangiogenic effects *in vivo*, this impact is only partial, and tumors continue to grow in spite of daily treatment (Al-Nedawi et al., 2009). It is also noteworthy that the loss-of-function mutations affecting pathways known to contribute to cellular vesiculation, such as TSAP6 (Yu et al., 2006; Lespagnol et al., 2008) and acidic sphingomyelinase (Asmase; Garcia-Barros et al., 2003; Bianco et al., 2009), do not prevent formation of spontaneous brain tumors in mice injected with oncogenic vectors (Meehan and Rak, unpublished observation). Furthermore, the loss of p53 suppressor, which regulates exosome formation through the TSAP6 activity at least in some settings (Yu et al., 2006; Lespagnol et al., 2008), in other models promotes tumorigenesis and EV-mediated shedding of tissue factor by cancer cells (Yu et al., 2005). Similarly, silencing of small GTPases involved in exosome biogenesis (e.g., Rab27a; Ostrowski et al., 2010), markedly affects melanoma metastasis in mouse models, but only modestly influences primary tumor growth (Peinado et al., 2012). In a recent study the antimetastatic effects of Rab27a manipulations varied considerably between different breast cancer cell lines revealing a rather “idiosyncratic” effect of this vesiculation pathway (Bobrie et al., 2012). Thus, specific cancers and the various processes involved in their distinct pathogenetic trajectories may differ with respect of the involvement of EVs.

It is possible that the contribution of EVs to cancer progression, or to the surrounding morbidity and mortality, could also be affected by other factors, such as host cell properties, host genetic background, cancer co-morbidities, medication, hormonal influences, inflammation, and other modifiers, which are presently poorly defined.

## Diagnostic Opportunities Associated with Cancer Cell Vesiculation

Accessibility of cancer-related EVs in biofluids, such as plasma, lymph, cerebrospinal fluid, urine, or malignant ascites or secretions brings to the fore some truly unprecedented diagnostic opportunities. For example, the analysis of the molecular cargo associated with circulating oncosomes may provide a unique, remote, non-invasive, and virtually continuous access to the changing molecular make up of cancer cells (virtually a “liquid biopsy”), with significant practical implications. This is a captivating prospect, as cancer cells themselves are often physically inaccessible, or cannot be frequently sampled and molecularly analyzed for obvious technical or ethical reasons.

In this context, the information encapsulated in the EV cargo (or present on the EV surface) may be especially relevant for molecular diagnosis of specific cancer subtypes, including molecular profiling and detection of driver mutations and putative drug targets. This could be achieved at the protein (Al-Nedawi et al., 2008; Graner et al., 2009; Shao et al., 2012), mRNA (Skog et al., 2008), or DNA levels (Balaj et al., 2011). Moreover, EVs represent a natural mechanism of molecular multiplexing, whereby they are potentially useful to extract information related to co-expression of various molecules in parental cancer cells. This could be useful to extract complex multimolecular classifiers of specific disease states, therapeutic responses, and clinical features, including proteomic, transcriptomic, and miR-based signatures (Skog et al., 2008; Taylor and Gercel-Taylor, 2008; Table 1).

**Table 1:** Possible applications of extracellular vesicles in cancer diagnosis and therapy*.

Potential application	Rationale and Hypothetical Effects	Relevant literature*
**EXTRACELLULAR VESICLES AS A PUTATIVE DIAGNOSTIC PLATFORM IN CANCER – EXAMPLES**		
EVs as diagnostic biomarker	Molecular diagnosis of cancer subtypes (e.g., marker or multiplex analysis of EV cargo, derivation of molecular signatures, involving proteins, mRNA, microRNA, and DNA)	Al-Nedawi et al. (2008), Graner et al. (2009), Shao et al. (2012)
	Correlative diagnostic classifiers (e.g., through developing inventories of diagnostic proteins, nucleic acids, lipids associated with the EV fraction)	Taylor and Gercel-Taylor (2008)
	Detection of circulating EVs containing epithelial markers (e.g., EpCAM and other molecules normally not found in plasma are often associated with tumor-derived EVs and can serve as a biomarker of cancer)	Taylor et al. (2011)
EVs as prognostic biomarker	Detection of cell-associated molecular markers related to disease aggressiveness (e.g., expression of oncogenic BRAF, MET, K-ras)	Al-Nedawi et al. (2010), Ramachandran et al. (2011), Peinado et al. (2012)
	Prognostic signatures (e.g., detection of protein and nucleic acid profiles associated with specific disease outcomes)	Poste and Nicolson (1980), Taylor and Gercel-Taylor (2008), Taylor et al. (2011)
EVs as predictive biomarker	Detection of actionable drug targets (e.g., EGFRvIII for the related vaccine; HER-2 for trastuzumab; BRAF for vemurafenib)	Koga et al. (2005), Al-Nedawi et al. (2008), Flaherty et al. (2012)
	Changes in the levels, nature, state, and composition of drug targets, or their modifiers (e.g., P-EGFR and PTEN in glioma)	Al-Nedawi et al. (2008), Putz et al. (2012)
	Signatures of cancer cell and cancer stem cell states relevant to therapy (e.g., indicators of signaling events, stemness, EMT, hypoxia, metabolic alterations)	Marzesco et al. (2005), Garnier et al. (2012), Muller (2012)
EVs as drug activity biomarker platform	Detection of drug target responses to therapeutics (e.g., activated/mutant states of oncoproteins, changes in levels of related phosphoproteins, gene expression signatures related to target inactivation or escape)	Al-Nedawi et al. (2008, 2010), Gonzales et al. (2009)
	Detection of correlative markers associated with cellular responses to drug exposure (e.g., protein levels, markers of stress response, activation of apoptotic pathways)	Muller (2012)
EVs as a pharmacodynamic and pharmacokinetic biomarkers	Markers of drug-target interactions (e.g., drug-target complexes, the presence of the drug in the EV cargo, as a reflection of drug-cell interaction; changes down-stream of the expected drug activity, Drug half-life in EVs versus plasma)	Zhuang et al. (2011), El-Andaloussi et al. (2012)
EVs as indicators of resistance to specific anticancer agents	High levels of circulating EVs containing drug targets and their modifiers (HER-2-EVs may act as both antagonist and indicators of reduced efficacy of trastuzumab; the content of HER-3 in breast cancer EVs may suggest resistance to HER-2 inhibitors)	Ciravolo et al. (2012)
	Mutant forms of drug targets (e.g., mutations of EGFR, such as L858R or T790M in circulating EVs may suggest either sensitivity or resistance to EGFR inhibitors, respectively; EV-associated mutant K-ras in colon cancer could be linked to resistance to cetuximab)	Linardou et al. (2009), Siena et al. (2009)
	Target multimerization (e.g., expression patterns of EGFR/HER related receptors in EVs may be suggestive of changing responses to EGFR or HER-2 antagonists)	Ritter et al. (2007)
	Detection of multidrug resistance markers and mediators (e.g., EVs may contain ABC transporters and other mediators of resistance to conventional chemotherapy)	Jaiswal et al. (2011)
Host-derived EVs as indicators of changes in the tumor microenvironment, immunity, and metabolism	Changes in levels of immune cell-derived EVs (e.g., exosomes may inform as to the emerging immunosuppression)	Andre et al. (2004)
	Presence of tumor antigens on host EVs and evidence of their presentation (e.g., exosomes emanating from dendritic cells could be informative as to the state of antitumor immunity; cancer-related exosomes may also have immune-suppressive activity)	Taylor and Gercel-Taylor (2005), Yang and Robbins (2011)
	Possible evidence of macrophage polarization and bone marrow cell recruitment (e.g., macrophage-related EVs may carry information as to the prevalence of M1 or M2 macrophages)	Qian and Pollard (2010)
	Possible detection of endothelial cell activation, damage, or death (e.g., in the context of antiangiogenic therapy endothelial EVs may alter their numbers, properties, and molecular composition; changes in VEGFR phosphorylation, IL-6 or IL-8 content, and other features in endothelial EVs may serve as biomarker of resistance to antiangiogenesis)	Diamant et al. (2004)
	EV-associated stromal determinants of cancer progression (e.g., stromal-derived exosomes may reflect composition of the cellular microenvironment in cancer subtypes, and be suggestive of disease aggressiveness; they may also reflect changes in the physical microenvironment (hypoxia), and be informative as to therapeutic responses, angiogenic activity, and other characteristics)	Finak et al. (2008)
EVs as indicators of cancer associated syndromes	EV-associated prothrombotic activities (e.g., circulating tissue factor containing EVs may be predictive of cancer coagulopathy or disease aggressiveness)	Sartori et al. (2011), Zwicker et al. (2011), van Doormaal et al. (2012)
	EV-associated mediators of normal tissue toxicity (e.g., EVs could carry markers of cardiomyocyte damage, and reflect other toxic side effects that may occur during anticancer therapy; EV-associated miRs and other molecules may correlate with the state of affected tissues)	Fichtlscherer et al. (2011)
**EXTRACELLULAR VESICLES AS A PUTATIVE THERAPEUTIC TARGET AND TOOL IN CANCER – EXAMPLES**		
Therapeutic blockade of EV production by cancer cells	Interference with molecular pathways of exosome biogenesis (e.g., silencing of Rab27a/b, neutral sphingomyelinase, and other pathways)	Trajkovic et al. (2008), Ostrowski et al. (2010), Peinado et al. (2012)
	Pharmacological blockade of ectosomal pathways (e.g., targeting floppases, sphingomyelinases, Rho, Arf6, AKT)	Bianco et al. (2009), Verderio et al. (2012)
Therapeutic blockade of EV production by host cells	Inhibition of myeloid cell vesiculation (e.g., FTY720 and other similar acting agents may influence the EV-mediated components of neuroinflammation, which may be relevant to the progression of brain tumors; similar effects could be applicable to other cancers)	Verderio et al. (2012)
	Inhibition of vesicular interactions within the angiogenic cell compartment (e.g., targeting endothelial exosomes may prevent angiogenic interactions between progenitor cells and mature endothelial cells)	Deregibus et al. (2007)
Therapeutic blockade of EV uptake	Blockade of surface PS residues with Annexin V analogs and other agents (e.g., Diannexin and similarly acting agents may prevent EVs from interacting with surfaces of target cells)	Al-Nedawi et al. (2009)
	Blockade of PS receptors on recipient cells (e.g., similar approaches as above could conceivably also change the surface properties of recipient cells preventing them from interacting with EVs)	Zhou (2007)
	Blockade of other receptors involved in EV-cell interactions (e.g., PSGL1 is responsible for interactions between EVs (particles) and cellular P-selectin. These processes can be antagonized pharmacologically with antibodies and other agents)	Furie and Furie (2004)
Targeting elements of the EV cargo	Antimirs directed at microRNA involved in intercellular communication (e.g., antagonizing cellular miRs would be expected to deplete them from the EV fraction)	Zhang et al. (2012)
	Kinase inhibitors (e.g., inhibitors of EGFR can impede the consequences of EV-mediated EGFR transfer between cells)	Al-Nedawi et al. (2008)
Elimination of tumor-related EVs from the circulation	Medical devices can be used to eliminate cancer derived exosomes from the circulating blood	Marleau et al. (2012)
Generation of EVs with pathway antagonistic activity	Interference with signaling pathways by presenting ligands or receptors on the surface of EVs (e.g., exosomes harboring Dll4 alter Notch signaling in recipient cells and could modulate tumor angiogenesis)	Sheldon et al. (2010)
EVs as drug delivery systems	Exosomes can be engineered to carry therapeutic molecules across tissue barriers (e.g., EVs can introduce siRNA into brain cells leading to gene downregulation)	Alvarez-Erviti et al. (2011), Zhuang et al. (2011), El-Andaloussi et al. (2012)
	EVs may potentially serve as vehicles to deliver tumor suppressors to cancer cells (e.g., suppressor microRNA, mRNA, and proteins may be delivered to target cells as cargo of exosomes; horizontal transfer of PTEN may serve to oppose cellular transformation)	Putz et al. (2012)
EVs as a cancer vaccine platform	Dendritic cell-derived exosomes may be used as cell-free cancer vaccine (e.g., dendritic cells may produce exosomes with the ability to present cancer antigens while being devoid of the risks and problems associated with manipulating viable or attenuated cancer cells)	Andre et al. (2004)

*These are presently largely theoretical possibilities, as no approved cancer therapy currently uses EVs as a tool, target, or companion diagnostic. The references do not provide evidence for these hypothetical scenarios, but merely point to the literature that may be relevant, or suggestive of a potential mechanism that may lead to EV use, development or feasibility of the respective approaches, as listed in the table.

In some instances the co-expression of molecules within the same cells may have significant therapeutic consequences and herald sensitivity or resistance to given treatments, e.g., co-expression of EGFRvIII and PTEN in glioma (Mellinghoff et al., 2005). EVs may be especially attractive in this context as they could preserve these combinatorial links. This could be particularly meaningful if EV samples could be used to detect the changing expression of putative drug targets, effectors of drug resistance, mediators of cancer-related co-morbidities, and paraneoplastic syndromes. These features may have both prognostic and therapeutic significance (Table 1). For example, there is an emerging interest in the role of EVs in assessing the risk of cancer patients to develop thrombotic disorders (coagulopathy). In some (but not all) cases this condition has been linked to the increase in plasma levels of tissue factor-bearing EVs and studies are underway to verify the applicability of this approach (Sartori et al., 2011; Thaler et al., 2012). Similar opportunities could also exist with regards to monitoring cancer cachexia, toxic organ damage, or inflammation (interleukins).

Amongst the most intriguing features of oncosomes is that, at least to some extent, they preserve the integrity, post-translational state and activation of oncogenic and regulatory proteins. This may facilitate detection of the effects exerted by biological agents directed against specific oncogenic targets, or as mentioned earlier, signal the emerging resistance to targeted therapy. For example, in animal models the phosphorylation status of the EV-associated and tumor cell-derived EGFR (P-EGFR) can be detected in plasma using a simple ELISA assay. Interestingly, P-EGFR levels change in response to EGFR inhibitors, and in concert to anticancer responses observed in these animals (Al-Nedawi et al., 2010).

At least in theory, EVs may also provide valuable information as to other post-translational modifications of key cellular proteins, as well as valuable sequence data indicative of either pre-existing, or new mutations within those molecules that may serve as therapeutic targets. Such mutations may be informative as to sensitivity or therapeutic resistance to certain drugs (e.g., EGFR inhibitors), molecular progression of the disease on, or off therapy, or could be related to the onset of an invasive phenotype. Interestingly, molecular EV cargo shifts markedly during epithelial to mesenchymal transition (EMT), or changes as a function of cellular stemness, and these changes may have biomarker utility (Marzesco et al., 2005; Garnier et al., 2012).

Tumor-related EVs circulating in blood are relatively numerous (even if diluted by EVs emanating from host cells), and represent either the whole cancer cell population, or its segments that have a sustained access to blood supply. As mentioned earlier, the EV content is protected from degradation and external influences, and remains relatively stable even upon storage. In principle, these features would separate EV based biomarkers from those associated with circulating tumor cells (CTCs), as these cells are rare and derived mainly from cancer cell subsets with a frank ability to intravasate. Thus, considerable advantages may exist in exploring the potential of EVs as a cancer biomarker platform.

While diagnostic exploration of EVs has attracted significant attention, the related technical challenges are also formidable (Figure 1). Tumor-derived EV often constitute a small minority amongst the circulating host EVs (<1%), and their contribution may vary depending on tumor type, size, and barriers of entry into different fluid spaces, as well as their unique and variable half lives in the circulation (Wang et al., 2012). In this regard new technologies of EV isolation, purification, and cargo analysis are being rapidly developed, including high sensitivity PCR techniques, such as BEAMing (Noerholm et al., 2012), new molecular profiling platforms (Kosaka et al., 2010), and multiplex microfluidics approaches with ultrasensitive detector systems based on nuclear magnetic resonance (NMR; Shao et al., 2012; van Doormaal et al., 2012). Indeed, several theoretical possibilities of using EVs as reservoirs of cancer biomarkers are under consideration (Table 1). While promising and attractive, these approaches are also complex and still poorly supported by mechanistic knowledge of the vesiculation processes in various cancer settings. Consequently, the impact of this field on clinical practice may still be years away.

## Therapeutic Potential of Extracellular Vesicles in Cancer

There are several, still largely theoretical, possibilities of exploiting the vesiculation process for the purpose of anticancer therapy. For example, in contexts where EV trafficking has a documented role in tumor formation, angiogenesis or metastasis, targeting pathways of EV production may hold promise. This notion has been explored through targeting Rab27a and sphingomyelinases in various settings, with encouraging but also often variable, results (Bobrie et al., 2012; Peinado et al., 2012; Verderio et al., 2012).

Likewise, blocking the EV uptake pathways may be worthy of more thorough consideration. Such an effect could be achieved by obliterating the relevant receptors on the surface of either tumor-derived EVs (e.g., PS), or the recipient cells to prevent the respective contact and merger. Alternatively, the interference with processes of EV pinocytosis could be considered as a way to achieve similar effects. Some of these possibilities have already been explored in aforementioned experiments with Annexin V analogs. Even though these agents are able to efficiently cloak PS residues *in vitro*, and are well tolerated *in vivo*, their antitumor effect is limited (Al-Nedawi et al., 2009). Perhaps the most impressive in this regard are the previously described studies on metastatic melanoma, where targeting Rab27a led to a significant suppression of metastatic niche effects, albeit with only a modest impact on the growth of primary tumors (Peinado et al., 2012). Once again, this example may suggests that the rate limiting role of EVs may be more pronounced against the metastatic disease then in the context of bulky tumors. Therefore it is conceivable that adjuvant use of EV antagonists may prevent the dissemination of cancer cells and expansion of dormant tumor foci.

It should be mentioned that cellular vesiculation could be therapeutically attractive for several additional reasons (Figure 1; Table 1). For example, exosomes are being explored as cancer vaccines (Andre et al., 2004), or as possible carriers of biological therapeutics. The latter avenue created understandable excitement because of the ability of engineered exosomes to pass through the blood brain barrier (BBB). To achieve this objective Alvarez-Evriti and colleagues devised a clever and effective method involving mast cell-derived exosomes manipulated to express brain penetrating 29-mer peptide (RVG) fused to Lampb2 vesicular membrane protein. Such exosomes equipped with a molecular key to unlock the BBB were used as vehicles to deliver anti-BACE1 siRNA to either cultured cells or brains of mice, in both cases resulting in a significant and specific target gene knockdown (Alvarez-Erviti et al., 2011; Ohno et al., 2012).

In another study the clinically approved sphingosine analog (FTY720) was recently shown to attenuate EV-mediated neuroinflammation (Verderio et al., 2012). These and other emerging examples illustrate how the biology of vesiculation may be eventually translated to the realm of anticancer therapy (Table 1). Indeed, targeting the cancer ‘ecosystem’ may require measures beyond targeting individual ‘species’ of cancer cells.

## Summary

It could be argued that the next major leap in our understanding of human cancer(s), and in gaining a better therapeutic control over these diseases (in their spectacular diversity), could come from studies on what occurs *between* cancer cells and not only *within* them. Oncogenic pathways not only alter the intracellular microenvironment, but also play a role in formation of aberrant intercellular communication milieu. This involves the impact of mutant genes on cancer cell secretome, but also on their vesiculation patterns and the ability to communicate by molecular exchange with their wider surroundings (cancer cell interactome). It is striking that some of the cancer-driving mutations persist only under certain external conditions and disappear from cultured cells.

Extracellular vesicles are a fascinating and unique part of this multicellular dynamics. They play still poorly defined pathogenetic roles but their targeting in cancer is of considerable interest. However, the ability of cancer (and stromal) cells to vesiculate is already being explored as a unique and natural mechanism to remotely reveal the complexity of molecular anomalies associated with human cancers. In this regard, EVs are the likely carriers of what is often described as circulating “cell-free” nucleic acids in plasma (cfNAs; microRNA and DNA), something that has long attracted diagnostic interests (Schwarzenbach et al., 2011). It is of interest to explore whether this could be extended to methylation profiles and studies on events affecting chromatin architecture. Using the EV fraction rather than total plasma in these studies could offer a significant enrichment opportunity, and enable detection of rare mutant sequences, other alterations, and their cell-specific combinations.

Cargo of circulating EVs may also provide clues, as to the representation of specific cell types (e.g., stem cells), and other aspects of tumor cell heterogeneity. The emerging approaches of quantitative proteomics (e.g., MRM analysis) may extend these opportunities even further and provide insights into the post-translational events involved in disease progression (e.g., molecular signaling states).

It should be noted, however, that major challenges do exist in all these areas, and the efficient extraction of molecular information from highly diluted and heterogeneous EV isolates presents a formidable technological barrier. Still, the recent developments are a source of considerable excitement and promise both in terms of new technologies and new concepts. Indeed, EVs epitomize the role of intercellular “computing” in complex diseases, such as cancer, and they inspire the interest in myriads of other processes that may underlie the interactomes of individual human tumors.

## Conflict of Interest Statement

Janusz Rak is a party in the patent application and the licensing agreement between McGill University and NXPharmaGen, in relation to microvesicle-related cancer diagnostics.
